# Reproductive Span of *Caenorhabditis Elegans* is Extended by *Microbacterium* Sp.

**DOI:** 10.2478/jofnem-2022-0010

**Published:** 2022-05-10

**Authors:** Tho Son Le, Thi Hong Gam Nguyen, Bich Hong Ha, Bui Thi Mai Huong, Thi Thu Hang Nguyen, Kim Dung Vu, Tu Cuong Ho, John Wang

**Affiliations:** 1 College of Forestry Biotechnology, Vietnam National University of Forestry, Hanoi, Vietnam; 2 Institute of Environmental Technology, Vietnam Academy of Science and Technology, Hanoi, Vietnam; 3 Biodiversity Research Center, Academia Sinica, Taipei, Taiwan

**Keywords:** bacteria, brood size, *Caenorhabditis* nematodes, lifespan, *Microbacterium* sp., physiology, *Protorhabditis* sp., reproductive span

## Abstract

The reproductive span (RS) of organisms could be affected by different factors during their lifetime. In the model nematode, *Caenorhabditis elegans*, RS is affected by both genetic and environmental factors. However, none of the factors identified so far were related to environmental bacteria, which may incidentally appear anywhere in the habitats of *C. elegans*. We aimed to find environmental bacteria that could affect the RS of *C. elegans* and related species. We tested 109 bacterial isolates and found that *Microbacterium* sp. CFBb37 increased the RS and lifespan of *C. elegans* but reduced its brood size. We studied the effect of *M*. sp. CFBb37 on the RS of *Caenorhabditis briggsae*, *Caenorhabditis tropicalis*, and another Rhabditidae family species, *Protorhabditis* sp., and found similar trends of RS extension in all three cases, suggesting that this bacterial species may induce the extension of RS broadly among *Caenorhabditis* species and possibly for many other Rhabditidae. This work will facilitate future research on the mechanism underlying the bacterial extension of RS of nematodes and possibly other animals.

*Caenorhabditis elegans* is a free-living nematode species that feeds primarily on bacteria (Darby, 2005; Samuel et al., 2016; Schulenburg and Felix, 2017). In the laboratory *C. elegans* is commonly fed *Escherichia coli* OP50, but this is not its natural food (Stiernagle, 2006). Previous studies of the habitats where *C. elegans* are found, such as soil, and rotting fruits and leaves, have found a diverse set of associated microorganisms, including fungi and bacteria (Dirksen et al., 2016; Zhang et al., 2017a; Dirksen et al., 2020; Stuhr and Curran, 2020; Zimmermann et al., 2020). Among these, the dominant taxa are *Proteobacteria*, *Bacteroidetes*, *Firmicutes*, and *Actinobacteria* (Samuel et al., 2016; Zecic et al., 2019). Although some of the associated microbes are detrimental to *C. elegans*, such as through the production of toxins (Gravato-Nobre and Hodgkin, 2005; Pukkila-Worley and Ausubel, 2012; Kumar et al., 2020), many of the bacterial species can serve as food.

Food quality and composition can affect animal physiology. Several studies have described the influence of bacterial diets, both of different *E. coli* strains and other species, on the growth and development rates, lifespan, and reproduction of *C. elegans* (Darby, 2005; Gracida and Eckmann, 2013; MacNeil et al., 2013; Frezal and Felix, 2015; Samuel et al., 2016; Zhang et al., 2017b; Kumar et al., 2020; Stuhr and Curran, 2020). Lifespan effects can be through nutritional differences among the different bacteria or the products of bacterial metabolism such as folate metabolism or nitric oxide production (Clark and Hodgkin, 2014; Kumar et al., 2020). With respect to reproduction, worms fed with *Comamonas* DA1877, *Methylobacterium*, or *Sphingomonas* bacteria had smaller brood sizes relative to OP50, while those fed with *Xanthomonas* bacteria or various other *E. coli* strains had comparable brood sizes (MacNeil et al., 2013; Stuhr and Curran, 2020). There is also evidence that bacterial noncoding RNAs can affect *C. elegans* physiology and behavior (Liu et al., 2012; Kaletsky et al., 2020).

One area that has little been studied is the effect of bacterial diets on the reproductive span (RS) of *C. elegans*. RS is defined as the duration in which a living organism has reproductive ability and is closely related to the concept of reproductive aging (Luo et al., 2009; Templeman and Murphy, 2018). In *C. elegans*, a recent study observed reduced RS when worms were fed with *Methylobacterium* or *E. coli* HB101 (Stuhr and Curran, 2020), while another study found extended RS when fed with *Staphylococcus epidermidis* (Madhu et al., 2019). In these studies, *E. coli* is a gut bacteria, *Methylobacterium* was isolated as a lab contaminant (Stuhr and Curran, 2020), and *S. epidermidis* is typically part of the human skin flora, so these bacteria are unlikely natural food for *C. elegans*. Thus, an open question is whether bacterial species that are natural food for *C. elegans* could affect RS. Decreases in RS may simply reflect deleterious interactions (e.g., toxins), however, a particularly interesting and less easily explained effect would be an increase in RS. This latter scenario may imply that some microbe–nematode interactions go beyond predator–prey, hinting at greater inter-taxa ecological complexity.

At the genetic level, RS is regulated by two signaling pathways. The first is the insulin/IGF-signaling (IIS) pathway. Mutations in *daf-2* (which encodes the IIS receptor tyrosine kinase) can extend RS (Hughes et al., 2007). Second, mutations of genes within the Transforming Growth Factor-β (TGF-β) Sma/Mab signaling pathway also extend RS, at least in part through the prolonged production of healthy oocytes (Luo et al., 2009; Templeman and Murphy, 2018). It is unknown whether bacteria would affect RS through these or other genetic pathways.

In this study, we wanted to determine whether any environmental bacteria could extend the RS of *C. elegans* and, possibly, related species. Therefore, we first isolated 109 natural strains of environmental bacteria and tested their influence on the reproduction of *C. elegans*. We found that one bacterial strain, *Microbacterium* sp. CFBb37, strongly extended both lifespan and RS, but reduced the offspring number, of *C. elegans*. *Microbacterium* sp. CFBb37 also extended RS in three other Rhabditidae species (two other *Caenorhabditis* species and a *Protorhabditis* sp.), suggesting that the extension of RS by this strain may be general across Rhabditidae. As this was a modest screen, our results suggest that more environmental bacterial species may be found that can extend RS. Additionally, our identification of *Microbacterium* sp. CFBb37 provides a potential entry point to study the molecular-genetic mechanisms for RS extension in both the bacteria and *C. elegans*.

## Materials and Methods

### Worm strains and maintenance

*C. elegans* Bristol var N2; *C. tropicalis* (BRC20400) (Le et al., 2021); *C. briggsae* CFB233 (a new wild isolate); and *Protorhabditis* sp. CFB231 (a new wild isolate). Information on the new wild isolates is given in Table S1. Worms were cultured in a 19 °C ± 1 °C incubator, unless otherwise noted. Worm strains were grown on Nematode Growth Media (NGM) plates seeded with either the *E. coli* OP50 control or test bacteria. Bacteria were cultured in a modified Luria-Bertani (LB) Broth at room temperature overnight prior to seeding the plates.

### Chemicals and media

NaCl (Bio Basic Canada Inc., 7647-14-5), peptone (TM MEDIA, 1506), agar (TM MEDIA, 242M), nutrient agar (TM MEDIA, TM341), yeast extract (Bio Basic Canada Inc., G0961), cholesterol powder (Across Organics, 110190250), CaCl_2_ (Fisher, 10043-52-4), MgSO_4_ (Fisher, 10034-99-8), KH_2_PO_4_ (Merck, 7778-77-0), and K_2_HPO_4_ (Fisher, 7758-11-4). Modified LB media: 5 g of yeast extract, 1 g of nutrient agar, and 10 g of NaCl in 1 L of distilled water (Sambrook and Russell, 2001). NGM was prepared following the standard protocol (Stiernagle, 2006).

### Isolation of wild nematodes and environmental bacteria

*C. briggsae* CFB233 and *Protorhabditis* sp. CFB231 were isolated as previously described (Le et al., 2021) as part of a larger survey from Cat Tien National Park in northern Vietnam. In short, rotting vegetation was plated onto OP50-seeded NGM petri plates and gravid adult nematodes that resembled *C. elegans* were isolated. For each strain, the sexual system (e.g., male-female or self-fertile hermaphrodites) was determined by singling out several L4 female individuals onto OP50-seeded NGM plates and then checking for self-progeny. PCR and sequencing of a fragment of the 18S rDNA using the primers SSU18A and SSU26R (Barriere and Felix, 2006) were used to assign the species (or genus) of the strain.

Environmental bacteria were isolated as previously described (Le et al., 2021) from Cat Tien and Cuc Phuong (southern Vietnam) National Parks in November 2019. In brief, rotting vegetation was diluted 10,000-fold in sterile water and then a 100–200 μl aliquot was plated onto an LB-agar plate. Bacteria colonies were scored for four qualitative plate phenotypes: size (big, medium, or small), color (transparent, red, or strong yellow), thickness (thin or thick), and prevalence (majority or minority). If a plate qualitatively contained fairly uniform phenotypes, possibly represented by one species, then two random colonies were picked. However, if a plate showed diverse bacterial phenotypes, then one colony of each putative type was picked (which was usually three to four). Twelve bacterial strains were identified to the species (or genus) level based on comparing the PCR amplified 16S rDNA sequence (primers QUGP-Fn5 and QUGP-Rn2, Vingataramin and Frost, 2015) to the NCBI nr database by BLAST (Zhang et al., 2000; Morgulis et al., 2008).

### Characterization of the reproductive system of *Protorhabditis* sp. CFB231

Reproductive organ morphology was examined using a microscope (ZEISS, VMI 0560) equipped with differential interference contrast (DIC) optics. 4′,6-diamidino-2-phenylindole (DAPI) was used to stain nuclei, especially those of potential sperm, as follows. Young adults (*N* = 15) were placed into 20 μl of 100% methanol (in a 1.5-ml Eppendorf tube) and then incubated at 4 °C for 30 min. Next, filter paper was touched to the methanol liquid surface for ∼5 s to wick away most of the methanol. Then, 15 μl of 200 ng/ml DAPI was added into the tube and the worms were incubated at room temperature (approximately 25 °C) for 30 min. Filter paper was used to wick away most of the DAPI solution, and then a drop of water was added into the tube. DAPI stained worms were observed at 63× magnification with an inverted microscope (ZEISS, VMI 0560) equipped with a camera (Axiocam 105 color) and a fluorescent light source.

### Testing the effect of different bacteria on RS

Three experiments were conducted to test the effect of bacteria on RS. The first experiment was a screen to identify candidate bacteria that affected the RS of *C. elegans*. To do this, gravid worms grown on OP50-seeded plates were bleached to release eggs (Stiernagle, 2006), which were then hatched on bacteria-free NGM plates overnight. After, the fresh L1 stage worms (P0) were washed off each plate with distilled water and approximately 10–15 worms were placed onto control OP50-seeded and test bacteria-seeded NGM plates. Each plate was then observed daily. We recorded the first and last day when we observed eggs on the plate, which was usually starting on day 3 and ending sometime between days 7 and 10. RS was then the number of days with eggs being laid. Because this was a screen, the P0 were not transferred to new plates and the effect of bacteria on RS were qualitatively judged as “similar to” or “different from” the OP50 control. All 109 bacterial strains and the OP50 control were tested in one big batch. Bacteria candidates that potentially extended RS (*N* = 16 strains) were screened a second time in an identical fashion.

In the second experiment the effect of bacteria strain on RS was determined more precisely. Here, only four bacteria strains were tested: *Microbacterium* sp. CFBb37 (screen suggested extended RS); CFBb9 and CFBb17 (both no obvious effect on RS) and the control OP50. In this experiment, RS was determined for second-generation *C. elegans* exposed to a test bacteria species. Parents (P0) were grown on the test bacteria species (since the egg or L1 stage), possibly providing some sort of acclimatization. Adult P0 (*N* = 10) worms were then transferred to a fresh test bacteria-seeded (or control OP50-seeded) plate, and allowed to lay eggs (F1) at room temperature. After 1–3 h, all P0 worms were removed and then the 6–15 F1s were cultured as a pool on the same plate at 19 ± 1 °C. The numbers of replicate pools for each bacterial strain were CFBb9 (*N* = 4), CFBb17 (*N* = 4, CFBb37 (*N* = 3), and OP50 (*N* = 5). After reaching adulthood, F1's were transferred daily to new plates. RS for each pool was then the number of days (plates) with eggs (F2's); RS for each bacterial strain was the average days across the replicate pools. The number of F2 progeny was also counted (see brood size assay below).

The third experiment differed from the second experiment in three ways. First, only one F1 was placed on each plate to eliminate any potential confounding effects from having pools of F1s per plate. Additionally, only *Microbacterium* sp. CFBb37 (*N* = 40 replicates) and OP50 (*N* = 41) were tested. Finally, RS was determined differently. A day was considered to be positive for brood if a one-sample *t*-test of brood number was significantly greater than zero (i.e., *P* < 0.05).

### Brood size assays

Brood sizes were determined as previously described (Le et al., 2021) with minor modifications. In brief, eggs on each plate were allowed to hatch and develop until the L2–L4 stages. To facilitate counting, the F2 offspring were sometimes killed with mild heat, which preserved the worm's shape, by passing the plates briefly over the flame of an alcohol lamp. Depending on the experiment, total or daily brood sizes are reported. Brood sizes of test bacteria were compared with that of OP50 using the paired *t*-test.

### Lifespan assays

Lifespan assays were conducted as previously described (Le et al., 2021). In brief, gravid P0 worms acclimated to test or control OP50 bacteria (since the egg or L1 stage) were transferred to a fresh bacteria-seeded NGM plate and allowed to lay eggs (F1) for 2 h before being removed from the plate. The next day, 20 F1 L1 or L2 worms were transferred to a new test or control OP50 bacteria-seeded plate. The F1 were transferred to new plates every 1 or 2 days until they died. A worm was scored as dead if it did not move when tapped with a worm pick. Lifespan was analyzed using the log-rank (Mantel–Cox) test (Kassambara, 2020).

### Assaying the presence of eggs in the uterus

This assay was designed to simplify determining RS by looking for eggs directly in the uteri of the focal adult worms. In other words, it did not require looking for eggs on a plate (i.e., RS experiment one, above), which may be rare near the end of the RS period, or moving the parent worm each day and then waiting for progeny to hatch and develop. To do this, a generation of worms (P0) was first acclimated to the test bacteria or OP50. P0 eggs were obtained by sodium hypochlorite bleaching gravid adults (Stiernagle, 2006); the eggs were hatched into L1 larvae on unseeded NGM plates, and then 90–100 L1 were transferred to culture on test (or control OP50) bacteria-seeded NGM plates. Gravid P0 adults were bleached for F1 eggs, which were hatched on the test bacteria-seeded NGM plate overnight at 20 °C. The next day, 50 L1 were transferred onto a new test or OP50 bacteria-seeded NGM plate. Every day after reaching adulthood, groups of 10–20 F1 adults were placed on a glass slide containing one drop of water and then overlain with a glass coverslip (Shaham, 2006). The number of eggs within each adult (i.e., from both uteruses) was counted. Comparison of the egg counts between the test bacteria and OP50 were analyzed using a paired *t*-test. To determine RS on the pooled adults, a day was considered to be positive for eggs if a one-sample *t*-test of egg number was significantly greater than zero (i.e., *P* < 0.05). Due to insufficient F1 adults, the full RS was not determined for *C. briggsae*, *C. tropicalis*, and *Protorhabditis* sp. on CFBb37.

### General statistics

All statistical analyses were done using R software (R Core Team, 2017).

## Results

### Isolation and screening of bacteria affecting RS of C. elegans

We hypothesized that the different bacteria encountered and eaten by *C. elegans* in the wild could affect its physiology, and in particular RS. To this end, we first isolated bacteria from rotting vegetation, substrates from which nematodes would often be found, from two national parks in Vietnam. We isolated 64 bacterial strains from 50 sampling sites in Cat Tien National Park and 45 strains from 44 sites in Cuc Phuong National Park (Tables S2 and S3). These 109 strains were qualitatively characterized based on colony size, color, thickness, and prevalence on the original screening plate (Table S3).

**Figure S1:**
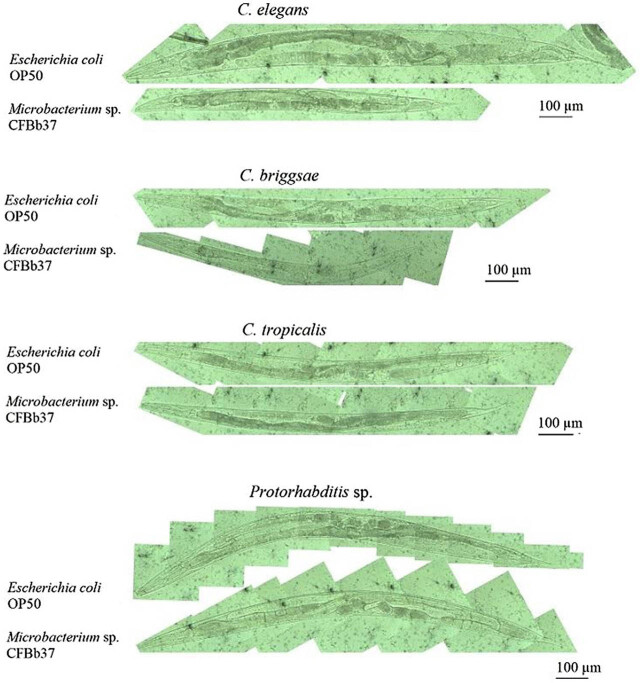
Size comparison of the four tested nematode species grown on *Microbacterium* sp. CFBb37 and *Escherichia coli* OP50. Images are representative samples.

Next, we used a simple 1-plate assay (see “Methods”) to screen all 109 isolates for an effect on RS of *C. elegans* in comparison to the *E. coli* OP50 control. The RS of *C. elegans* on OP50 was about 3–4 days. Qualitatively, 108 strains also had similar RS in the 3- to 4-day range. One strain, CFBb37, seemed to extend RS to approximately 7 days. We note that we found a nematode, *Caenorhabditis briggsae*, co-occurring in the field sample containing CFBb37 (Table S2). We also noticed that this and six other bacterial strains reduced the body size and another delayed the onset of first brood production in *C. elegans* (Fig. S1 and Table S2). To determine their genus or species identity we sequenced the 16S rDNA barcode for these eight bacterial strains as well as four others without obvious effects on *C. elegans* (Table S2). This revealed that CFBb37 is a *Microbacterium* sp., which is in the Actinobacteria phylum. Most of the other strains belonged to the phylum Proteobacteria (*n* = 8) while the rest were in the phyla Bacteroidetes (*n* = 1) or Firmicutes (*n* = 2). We focused on *Microbacterium* sp. CFBb37 for the rest of this study.

### Confirmation of RS extension by *Microbacterium* sp. CFBb37

To confirm the screening results, we determined more precisely the RS of *C. elegans* when fed with *Microbacterium* sp. CFBb37, the control OP50, and two additional identified strains (*Acinetobacter* sp. CFBb9 and *Serratia* sp. CFBb17). In this assay we moved pools of adults (*n* = 6–15) to fresh plates daily, which allowed determining both the presence of brood and counting the number of brood per day. The worms growing on OP50 as well as *Acinetobacter* sp. CFBb9 and *Serratia* sp. CFBb17 had similar RS durations, which was 4 days (*P* < 0.001; Table 1). In contrast, worms growing on *Microbacterium* sp. CFBb37 had an RS of 8 days (*P* < 0.001), confirming the RS extension from the screen. Qualitatively, average daily brood counts appeared to decline exponentially from day 2 onward (day 2, *n* = 47; day 7 and later, *n* < 4) (Table 1).

**Table 1:** Average daily individual brood counts^a^ for *C. elegans*on three environmental bacterial species and the OP50 control.

**Reproductive day number**	***Acinetobacter* sp. CFBb9 (*N* = 4, *n* = 29)^b^**		***Serratia* sp. CFBb17 (*N* = 4, *n* = 39)^b^**		***Microbacterium* sp. CFBb37 (*N* = 3, *n* = 37)^b^**		***Escherichia* coli OP50 (*N* = 5, *n* = 33)^b^**	
	**Eggs laid per individual**	**Greater than zero *P* (one-sample *t*-test)**	**Eggs laid per individual**	**Greater than zero *P* (one-sample *t*-test)**	**Eggs laid per individual**	**Greater than zero *P* (one-sample *t*-test)**	**Eggs laid per individual**	**Greater than zero *P* (one-sample *t*-test)**
Day 1	41.24	<0.001	69.30	<0.001	35.16	<0.001	151.69	<0.001
Day 2	162.34	<0.001	165.74	<0.001	47.00	<0.001	95.39	<0.001
Day 3	44.51	<0.001	39.23	<0.001	24.08	<0.001	2.96	<0.001
Day 4	2.07	<0.001	0.95	<0.001	13.59	<0.001	1.12	<0.001
Day 5	0		0		8.86	<0.001	0	
Day 6	0		0		6.16	<0.001	0	
Day 7					3.51	<0.001		
Day 8					1.49	<0.001		
Day 9					1.02	<0.001		
Day 10					0.24	<0.001		
Day 11					0.51	<0.001		
Day 12					0			
Day 13					0			
Average brood	250.16		275.22		141.62		251.16	
size per worm^c^								
RS (days)^d^	4		4		11		3.8	

a First, an average brood number per individual was calculated per pool, then the average of the pools was reported.

b N, number of pools tested; n, total numbers of individuals tested.

c Average brood size per worm in pools is the sum of all the daily brood sizes.

d RS is the number of days with average daily individual brood counts >0 based on one-sample *t*-test.

RS, reproductive span.

**Figure 1:**
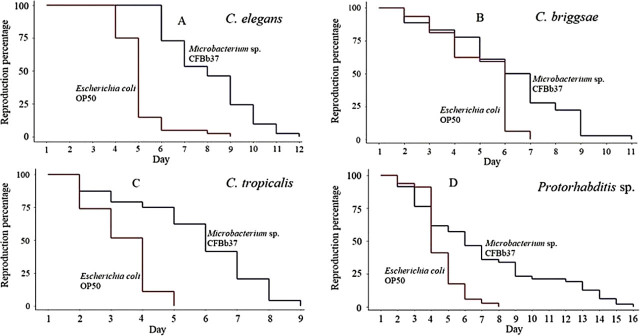
RS of individual nematodes on *Microbacterium* sp. CFBb37 and the *E. coli* OP50 control. (A) *C. elegans*, (B) *C. briggsae*, (C) *C. tropicalis*, and (D) *Protorhabditis* sp. on CFBb37 had longer RS than on OP50 (*P* < 0.001, log-rank test). Full RS data in Table 2. RS, reproductive span.

In the above assay, the number of parents per plate varied both within and among bacterial strains. While unlikely to be of major effect, we decided to eliminate the potential confounding effects of parental interactions and differing parental density by re-examining brood presence (and brood sizes, see below) using one parental worm per plate. We found that the average RS for worms growing on *Microbacterium* sp. CFBb37 was 79% greater than on OP50 (7.29 ± 0.25 days; mean ± 1 standard error (SE) versus 4.08 ± 0.16; *P* < 0.001; Fig. 1A and S1; Table 2). The combined data clearly indicate that CFBb37 induces RS extension in *C. elegans*.

**Table 2:** Total average brood and RS of tested nematode species.

**Reproductive day number**	**OP50**		**CFBb37**		**Different between the strains *P* (paired *t*-test)**
	**Eggs laid per day (mean ± SE)**	**Greater than zero *P* (one-sample *t*-test)**	**Eggs laid per day (mean ± SE)**	**Greater than zero *P* (one-sample *t*-test)**	
*C. elegans*					
Day 1	32.68 ± 5.04	<0.001	3.76 ± 1.74	<0.001	<0.01
Day 2	149.75 ± 4.71	<0.001	24.64 ± 2.53	<0.001	<0.001
Day 3	78.55 ± 6.46	<0.001	23.49 ± 2.12	<0.001	<0.001
Day 4	3.25 ± 0.81	<0.001	22.05 ± 2.31	<0.001	<0.001
Day 5	1.40 ± 0.77	>0.05	15.10 ± 1.75	<0.001	<0.001
Day 6	0.58 ± 0.55	>0.05	7.93 ± 1.14	<0.001	<0.001
Day 7	0.05 ± 0.03	>0.05	4.39 ± 0.79	<0.001	<0.001
Day 8	0.03 ± 0.03	>0.05	1.88 ± 0.51	<0.001	<0.01
Day 9	0		0.56 ± 0.23	<0.05	N.A.
Day 10			0.07 ± 0.04	>0.05	N.A.
Day 11			0.03 ± 0.03	>0.05	N.A.
Day 12			0		
*n* ^a^	40		41		
Average brood size per worm^b^	266.28 ± 6.42		137.63 ± 5.74		<0.001
RS (day)^c^	4.08 ± 0.16		7.29 ± 0.25		<0.001 (log-rank test)
*C. briggsae*					
Day 1	11.59 ± 1.33	<0.001	2.11 ± 0.34	<0.001	<0.001
Day 2	18.21 ± 1.89	<0.001	3.80 ± 0.57	<0.001	<0.001
Day 3	22.56 ± 3.46	<0.001	3.17 ± 0.56	<0.001	<0.001
Day 4	12.00 ± 2.52	<0.001	2.03 ± 0.50	<0.001	<0.001
Day 5	0.88 ± 0.28	<0.01	2.30 ± 0.50	<0.001	0.0109
Day 6	0.16 ± 0.10	>0.05	1.69 ± 0.44	<0.001	<0.01
Day 7	0		0.50 ± 0.17	<0.001	N.A.
Day 8			0.22 ± 0.07	<0.01	N.A.
Day 9			0.08 ± 0.08	>0.05	N.A.
Day 10			0.08 ± 0.08	>0.05	N.A.
Day 11			0		
*n* ^a^	32		36		
Average brood size per worm^b^	65.40 ± 7.09		16.00 ± 1.88		<0.001
RS (day)^c^	3.97 ± 0.25		5.61 ± 0.39		<0.001 (log-rank test)
*C. tropicalis*					
Day 1	4.37 ± 0.78	<0.001	4.75 ± 0.65	<0.001	1.0
Day 2	4.81 ± 1.37	<0.001	4.79 ± 0.92	<0.001	0.8652
Day 3	1.22 ± 0.39	<0.01	7.20 ± 1.71	<0.001	<0.01
Day 4	0.37 ± 0.24	>0.05	3.88 ± 1.05	<0.01	<0.01
Day 5	0		3.29 ± 0.92	<0.01	N.A.
Day 6			0.95 ± 0.29	<0.01	N.A.
Day 7			0.33 ± 0.14	<0.05	N.A.
Day 8			0.04 ± 0.04	>0.05	N.A.
Day 9			0		N.A.
*n* ^a^	27		24		
Average brood size per worm^b^	10.78 ± 1.90		25.25 ± 3.59		<0.01
RS (day)^c^	2.37 ± 0.20		4.96 ± 0.36		<0.001 (log-rank test)
*Protorhabditis* sp.					
Day 1	45.00 ± 5.02	<0.001	2.70 ± 0.30	<0.001	<0.001
Day 2	95.15 ± 7.18	<0.001	4.21 ± 0.49	<0.001	<0.001
Day 3	18.06 ± 4.95	<0.001	3.23 ± 0.52	<0.001	<0.01
Day 4	2.00 ± 0.69	<0.01	3.12 ± 0.56	<0.001	>0.05
Day 5	0.88 ± 0.70	>0.05	2.38 ± 0.43	<0.001	N.A.
Day 6	0.24 ± 0.18	>0.05	1.36 ± 0.28	<0.001	N.A.
Day 7	0.06 ± 0.06	>0.05	1.09 ± 0.24	<0.001	N.A.
Day 8	0		1.00 ± 0.24	<0.001	N.A.
Day 9			1.17 ± 0.33	<0.01	N.A.
Day 10			0.53 ± 0.18	<0.01	N.A.
Day 11			0.53 ± 0.19	<0.01	N.A.
Day 12			0.42 ± 0.14	<0.01	N.A.
Day 13			0.14 ± 0.06	<0.05	N.A.
Day 14			0.10 ± 0.07	>0.05	N.A.
Day 15			0.06 ± 0.04	>0.05	N.A.
			0		
*n* ^a^	34		47		
Average brood size per worm^b^	161.38 ± 10.05			22.08 ± 2.26	<0.01
RS (day)^c^	3.59 ± 0.20			6.68 ± 0.61	<0.001 (log-rank test)

a n, the total number of tested individuals in each test.

b Average brood size per worm was the total brood of all hermaphrodites (n) divided by n.

c Average RS. N.A., not applicable.

RS, reproductive span; SE, standard error.

### Daily count of eggs in the uterus of *C*. *elegans*

Our assays for RS above were sometimes difficult (e.g., looking for rare laid eggs near the end of the RS period) or required moving the worms to new plates everyday as well as waiting for the progeny to hatch. A simplification of the RS assay would be to directly look for eggs in the uteruses (“uterus egg assay”) of the focal adult worm at 400× magnification under a DIC compound microscope each day. The presence of at least one egg would indicate fertility. The lack of eggs could indicate reproductive senescence or that any eggs were recently laid. Examining many adult worms each day should help distinguish between the two possibilities, although there would still be a bias for incorrectly scoring a batch of worms (i.e., day) as reproductively senescent.

To test whether the uterus egg assay gives comparable results to our original RS assays, we placed *C. elegans* L1s on either CFBb37 or OP50 and raised them to adulthood. Then, each day we counted the number of eggs in the uterus of 29–37 worms. For each day, if this number of eggs was greater than zero (using a one-sample *t*-test), then we considered that day to be a fertile day (i.e., not reproductively senescent).

Using this assay, the worms on CFBb37 had an RS of 8 days while those on OP50 had an RS of 4 days (Table S5). The results from counting eggs in the uteruses were qualitatively concordant with the earlier brood-based assays (7.29 and 4.08 days, respectively; Table 2). Thus, although this approach has an inherent bias for reproductive senescence (i.e., scoring shorter RS), we suggest that the simpler uterus egg assay provides a reasonably good estimate for the RS, and can distinguish differences in RS, at least for those separated by several days.

### Reduction of brood size by *Microbacterium* sp. CFBb37

While confirming the RS extension by *Microbacterium* sp. CFBb37 on pools of *C. elegans*, we noticed that this bacterial strain reduced the average total brood size compared with OP50 (141.6 vs 251.2; *P* < 0.001; Table 1). As above, to remove the potential confounding effects of pooled worms, we recounted the brood sizes using one parental worm per plate. We found that the average total brood size on *Microbacterium* sp. CFB37 was less than on OP50 (137.63 mean ± 5.74 SE versus 266.28 ± 6.42; *P* < 0.001, Table 2). Partitioning the data into daily brood sizes over the first 8 days (i.e., the number of days with data for both bacterial strains, Table 2) revealed that worms fed with OP50 had greater brood sizes than those fed with *Microbacterium* sp. CFB37 during the first 3 days (29–125 more eggs/day) while the opposite (2–19 fewer eggs/day) occurred during the last 5 days (all *P* < 0.001, Table 2). Although there were more days where worms growing on CBFb37 had greater brood sizes than OP50, the magnitude of the differences was smaller during these 5 days, explaining the lower average total brood sizes for *Microbacterium* sp. CFB37.

### Lifespan extension by *Microbacterium* sp. CFBb37

**Figure 2:**
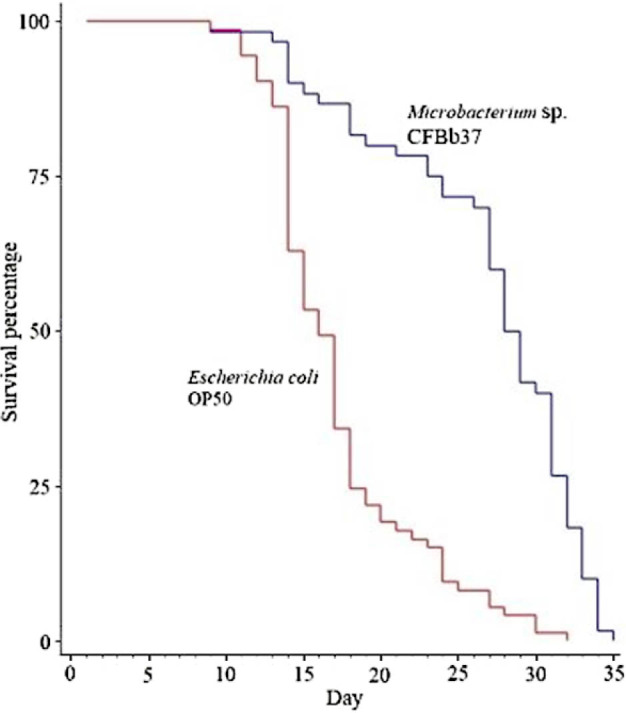
Lifespan assays of *C. elegans* on *Microbacterium* sp. CFBb37 and the OP50 control. *C. elegans* grown on CFBb37 survived longer than OP50 (*P* < 0.001, log-rank test). Detailed lifespan data are in Tables S4 and S5.

Bacterial diet has been shown to affect the (somatic) lifespan of *C. elegans* in many cases (Zhang et al., 2017; Kumar et al., 2020). Thus, we tested whether *Microbacterium* sp. CFBb37 could also alter the *C. elegans* lifespan. We found that worms fed with *Microbacterium* sp. CFBb37 lived longer than those fed with OP50 (26.77 ± 0.86 days, mean ± 1 SE, versus 17.26 ± 0.57; *P* < 0.001; Fig. 2 and Table S4).

To determine at what general “time period” lifespan extension was occurring, we partitioned the total lifetime into the time spent in the larval, reproductive, and post-reproductive periods. For *Microbacterium* sp. CFBb37, *C. elegans* spent an average of 2.89 days as larvae, 7.29 days as reproductive adults, and 16.59 days as post-reproductive adults. On OP50, these durations were 2.05, 4.08, and 11.13 days, respectively. Thus, worms fed with CFBb37 spent longer periods of time in all three stages. This suggests that the total lifespan of the worms on CFBb37 was likely a combination of slower growth as larvae plus lifespan extension during reproductive and post-reproductive stages.

### Extension of RS in closely related species

**Figure S2:**
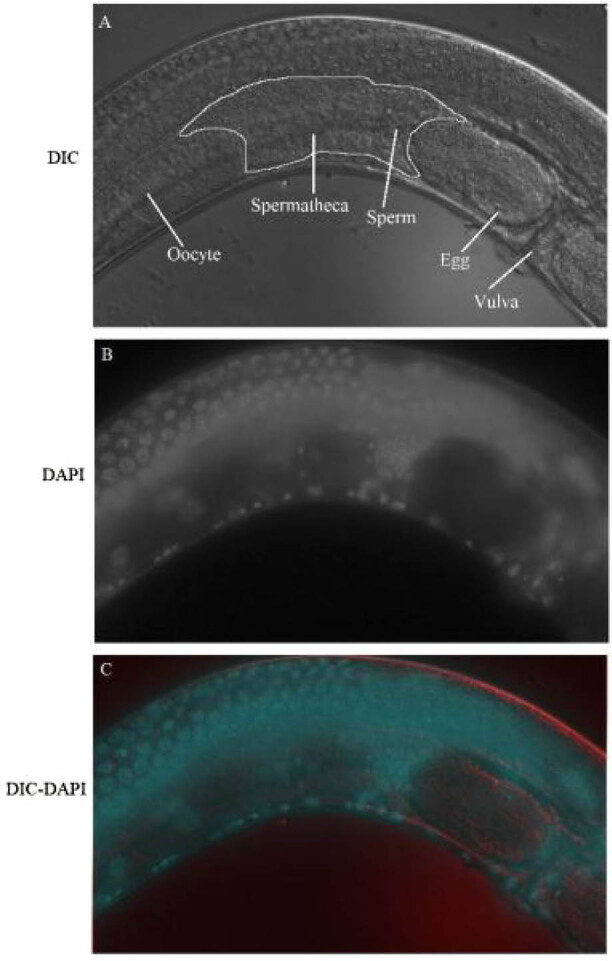
*Protorhabditis* sp. CFB231 make sperm. (A) DIC image of spermathecal region (outlined). One oocyte is apparently transiting the spermatheca. (B) DAPI staining for nuclei. (C) Merged images of DIC and DAPI. DIC, differential interference contrast.

The extension of RS by *Microbacterium* sp. CBFb37 (compared with *E. coli* OP50) in *C. elegans* could be a species-specific effect. Alternatively, *Microbacterium* sp. CBFb37 may also extend RS in other worm species. We tested this by examining RS using both the uterus egg assay and the presence of brood assay for two additional *Caenorhabditis* species (*C. briggsae* CFB233 and *C. tropicalis* BRC20400) and another Rhabditidae family species, *Protorhabditis* sp. CFB231. *C. briggsae* CFB233 and *Protorhabditis* sp. CFB231 were isolated from two different field samples that were each also the source of two bacterial strains (Tables S2 and S3). The sexual system of *Protorhabditis* sp. CFB231 could be self-fertile hermaphrodites, because their spermathecae contain sperm (Fig. S2). However, other parthenogenetic *Protorhabditis* species also produce sperm (Fradin et al., 2017; Grosmaire et al., 2019), so additional genetic analysis will be needed to determine the sexual system of this species.

For all three species and for both assays, we found that feeding on *Microbacterium* sp. BCFb37 extended the RS compared with OP50 (all *P* < 0.001, log-rank test; Figures 1B–D and S1; Tables 2 and S5). For *C. briggsae* the RS extension was 1.64 additional days (+41%), for *C. tropicalis* 2.59 days (+109%), and for *Protorhabditis* sp. 3.09 days (+86%). Together, these results suggest that *Microbacterium* sp. CBFb37 can likely extend RS broadly across the *Caenorhabditis* genus and perhaps, at least, across the Rhabditidae family.

### Brood sizes of closely related species on *Microbacterium* sp. CFBb37

The extension of RS in *C. elegans* by *Microbacterium* sp. CBFb37 was associated with a decrease in brood size. We next examined whether the extension of RS in the three other species was also associated with a consistent decrease in brood size. We found that the brood sizes of both *C. briggsae* (16.00 ± 1.88 *Microbacterium* sp. CBFb37 versus 65.40 ± 7.09 *E. coli* OP50; *P* < 0.001; Table 2) and *Protorhabditis* sp. (22.08 ± 2.26 versus 161.38 ± 10.05; *P* < 0.01; Table 2) on *Microbacterium* sp. CFBb37 was less than on *E. coli* OP50. However, *C. tropicalis* brood size on *Microbacterium* sp. CFBb37 was more than on *E. coli* OP50 (25.25 ± 3.59 versus 10.78 ± 1.90; *P* < 0.01; Table 2). Together, these results suggest that the interaction between *Microbacterium* sp. CFBb37 and the nematodes is not simple.

## Discussion

The primary goal of this study, and to the best of our knowledge, was to investigate the effect on RS by natural isolates of bacteria that are found in environments where nematodes can be typically found. From 109 wild bacterial isolates in Vietnam, we succeeded in finding one, *Microbacterium* sp. CFBb37, which extended RS in *C. elegans* as well as three other related species. RS extension, however, did not correlate with brood size in a consistent way with three species having fewer progeny (*C. elegans*, *C. briggsae*, and *Protorhabditis* sp.) and one with more (*C. tropicalis*).

Many studies have investigated various factors that reduce RS in *C. elegans*. While no environmental bacteria have been previously tested, one study did find that another *E. coli* strain and a laboratory bacterial contaminant reduced the RS of *C. elegans* (Stuhr and Curran, 2020). Similarly, worms cultured in axenic media (presumably with suboptimal nutrient conditions) (Croll et al., 1977) or at higher temperatures (Klass, 1977) also had reduced RS compared with the standard conditions.

Extension of RS has been observed in *C. elegans*. Worms grown on *S. epidermidis* (Madhu et al., 2019); at lower temperatures (Klass, 1977; Huang et al., 2004); in the presence of the pharmacological reagents, ethosuximide (Hughes et al., 2007) or metformin (Onken and Driscoll, 2010); and with the addition of vitamin E (Harrington and Harley, 1988) or trehalose (Honda et al., 2010), exhibited longer RS. Extended RS is also seen with some mutants such as *eat-2*, which causes dietary restriction, and in certain IIS pathway and TGF-β Sma/Mab genes (Huang et al., 2004; Hughes et al., 2007; Hughes et al., 2011). These RS extensions are from ∼12% to ∼116%. Greater RS extension (∼25% to ∼160%) was found for 32 genes in an RNAi screen (Wang et al., 2014). With the caveat that the setup of these and our experiments differed, the 79% RS extension we observed for *Microbacterium* sp. CFBb37 is larger than most of these factors or reduction-of-function phenotypes.

**Figure 3:**
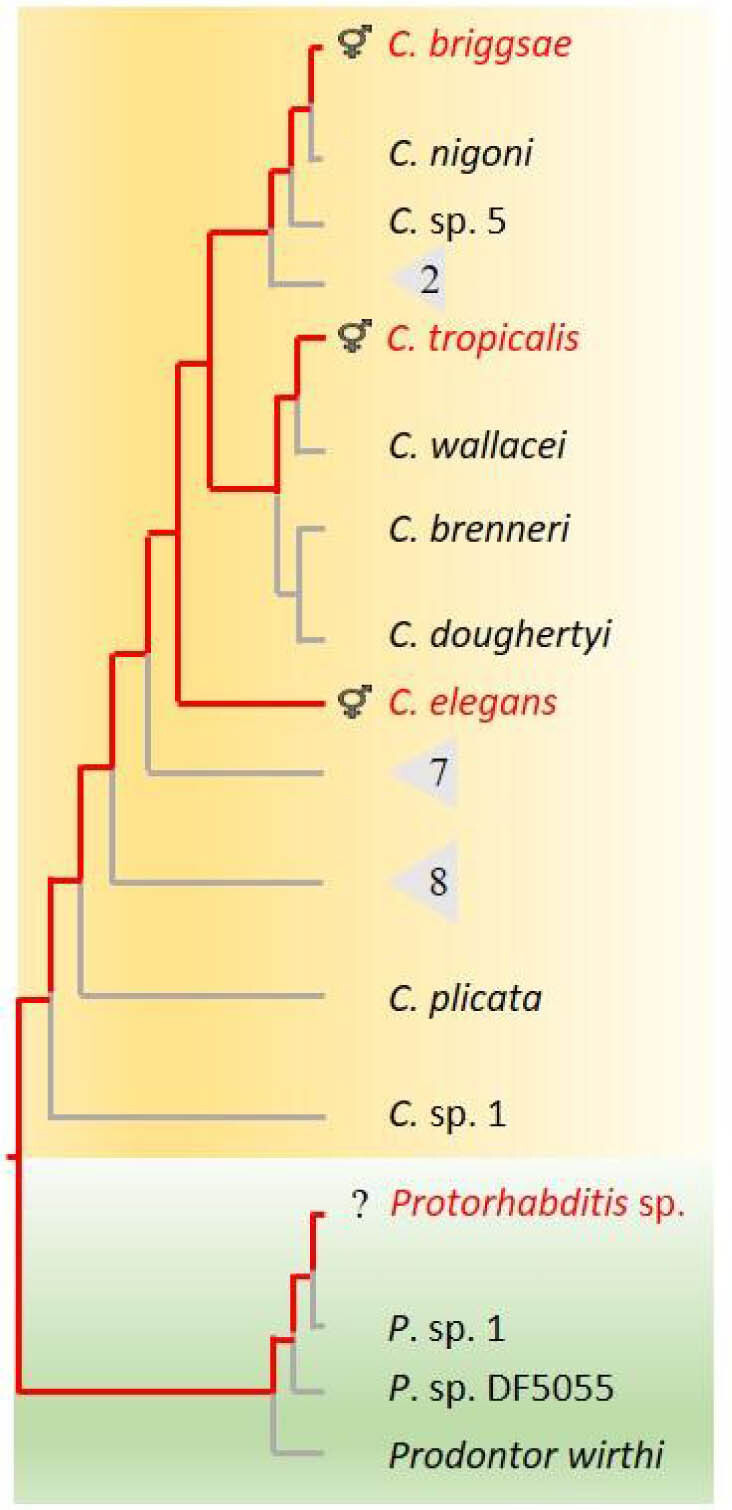
Summary of RS extension in the *Caenorhabditis* species (orange) and the outgroup *Protorhabditis* sp. (light blue). Red lines indicate inferred ancestral evolutionary lineages with extended RS. We suggest that other species within the genus *Caenorhabditis* are likely to have extended RS but this is untested. For simplification, some species are not listed and are grouped together in triangles, with the numbers of known species indicated. Phylogeny is adapted from Kiontke and Fitch (2005) and Felix et al. (2014) with the position of *Protorhabditis* sp. CFB231 placed next to *P*. sp. 1 species based on the 18S ribosomal RNA gene sequence (Zhang et al., 2000; Morgulis et al., 2008). *Protorhabditis* sp. CFB231 is self-fertile but the sexual system, hermaphroditic or parthenogenetic, is unknown (indicated by a question mark). RS, reproductive span.

As mentioned earlier, we found that *Microbacterium* sp. CFBb37 extended RS in two other *Caenorhabditis* species and a *Protorhabditis* sp. The magnitude of RS extension for these species was relatively large, ranging from 41% to 109%. *Protorhabditis* sp is part of a clade that is sister to the *Caenorhabditis* genus and together they are part of the “Eurhabditis” group. These results suggest that *Microbacterium* sp. CFBb37 could extend RS broadly among Eurhabditis, and possibly for many other Rhabditidae (Fig. 3). *Microbacterium* sp. CFBb37 was also co-isolated with *C. briggsae*, indicating that these two species likely encounter each other in natural settings. Overall, this leads us to suggest that this microbe–nematode interaction represents an ecologically relevant, and possibly important, interaction.

The mechanism by which *Microbacterium* sp. CFBb37 extends RS remains to be determined. Because RS extension occurs in several species, it likely operates through a conserved pathway. One possibility is dietary restriction, or starvation. Starved adults form elevated rates of bags of worms, or “worm bags”, often resulting in internal hatching of eggs (Angelo and Van Gilst, 2009). We observed very few worm bags on both *E. coli* OP50 and *Microbacterium* sp. CFBb37, suggesting that the RS extension we see is different from simple starvation. Another possibility is that *Microbacterium* sp. CFBb37 produces a presumably weak toxin, or alternatively provides less of a limiting nutrient. This could plausibly explain the slower larval development and reduced brood sizes, the latter of which might be the indirect consequence of reduced oocyte quality or compromised oogenesis (Kadandale and Singson, 2004; Templeman and Murphy, 2018; Templeman et al., 2020). Given the identification of many genes that increase RS, a third possibility could be that *Microbacterium* sp. CFBb37 somehow acts through one or more of these genes. Future studies will be needed to determine the molecular-genetic bases for *Microbacterium* sp. CFBb37-mediated RS extension.

In *C. elegans*, RS extension by *Microbacterium* sp. CFBb37 is associated with increased lifespan but fewer progeny compared with the standard *E. coli* OP50 (Table 2 and Table S4). This result is consistent with a frequently observed, apparent tradeoff between lifespan and progeny production (Maklakov and Immler, 2016; Scharf et al., 2021). However, there are exceptions to this negative relationship, including the composition of the cultivation media (Le et al., 2021). Our results with *C. tropicalis* are another example where the tradeoff does not always hold (Fig. 2; Table 2 and Table S4). Although a caveat is that brood sizes were low on both *E. coli* OP50 (∼11) and *Microbacterium* sp. CFBb37 (∼25), possibly indicating that *C. tropicalis* is not in a healthy state on either of these bacteria.

## Conclusions

We have found that *C. elegans* cultured on a bacteria species, *Microbacterium* sp. CFBb37, extended RS and lifespan but reduced brood size. Within the family Rhabditidae, two *Caenorhabditis* species and another relatively close species, *Protorhabditis* sp. also exhibited RS extension and altered progeny production. Additionally, *Microbacterium* sp. CFBb37 was co-isolated with *C. briggsae*. Together, these results suggest that *Microbacterium* sp. CFBb37 potentially prolongs RS and affects brood size generally in other Rhabditidae nematodes and this microbe–nematode interaction is likely ecologically relevant.

## Appendix

**Table S1:** Wild-type isolates of nematodes in Cat Tien National Park.

**Species name**	**Strain number**	**GenBank accession No.**	**Best BLAST hit^a^**	**Query coverage**	**Identity (%)**	**Bit score (max/total)**	**E value**
*Caenorhabditis briggsae*	CFB233	MZ457559	*Caenorhabditis briggsae* AF16	98%	100%	1,544/1,544	0.0
*Protorhabditis* sp.	CFB231	MZ474671	*Protorhabditis* sp. 1 GVDU-2019	90%	95.35	1,229/1,229	0.0

a Query date March 25, 2022.

**Table S2:** Bacterial strains isolated from rotting vegetation samples collected in Cat Tien National Park and their effects on nematodes.

**Strain^a^**	**Species**	**Isolation site**	**GenBank accession No.**	**Phenotype**
CFBb3	*Pseudomonas* sp.	11°23′54.4″N 107°20′21.4″E	OM456346	Late laying egg
CFBb9^b^	*Acinetobacter* sp.	11°24′20.9″N 107°24′22.3″E	MZ467312	Normal
CFBb11	*Acinetobacter* sp.	11°24′20.9″N 107°24′22.3″E	OM456348	Small body
CFBb17	*Serratia* sp.	11°24′21.4″N 107°24′21.4″E	MZ457333	Small body
CFBb35	*Serratia* sp.	11°27′5″N 107°27′25.6″E	OM629173	Normal
CFBb37	*Microbacterium* sp	11°27′5″N 107°27′25.6″E	MZ453404	RS; small body
CFBb39	*Sphingobacterium* sp.	11°27′17.9″N 107°22′5.6″E	OM892000	Normal
CFBb40	*Bacillus* sp.	11°27′17.9″N 107°22′5.6″E	OM456347	Normal
CFBb42	*Serratia* sp.	11°26′47.8″N 107°26′19.8″E	OM456350	Small body
CFBb53	*Bacillus cereus*	11°26′12.1″N 107°25′25.7″E	OM883917	Small body
CFBb54	*Enterobacter* sp.	11°26′12.1″N 107°25′25.7″E	OM456349	Small body
CFBb57	*Serratia* sp.	11°23′26″N 107°21′1.8″E	OM892003	Small body

Gray highlights bacteria isolated from the same sites as the two new nematode strains isolated from this study (*C. briggsae* CFB233 [light gray] and *Protorhabditis* sp. CFB231 [dark gray]).

a Strains were isolated in this study unless indicated.

b From Le et al., 2021.

RS, reproductive span.

**Table S3:** Bacterial isolates with description of colonial morphology, and geography of sample sites.

**Cat Tien National Park**				**Cuc Phuong National Park**			
**Strain**	**Morphology of colony^a^**	**North**	**East**	**Strain**	**Morphology of colony^a^**	**North**	**East**
CFBb1	Small	11°23′57.5″	107°20′21.4″	CFBb65	Big	20°17′40.4″	105°39′46.6″
CFBb2	Big	11°23′54.4″	107°20′21.4″	CFBb66	Big	–	–
CFBb3	Small	–	–	CFBb67	Small	–	–
CFBb4	Irregular	11°24′22.8″	107°24′22″	CFBb68	Small	–	–
CFBb5	Irregular	–	–	CFBb69	Big	20°17′40″	105°39′57.5″
CFBb6	Big	–	–	CFBb70	Small	–	–
CFBb7	Big	–	–	CFBb71	Small	20°17′41.3″	105°39′59.6″
CFBb8	Small	–	–	CFBb72	Small	–	–
CFBb9	Big	11°24′20.9″	107°24′22.3″	CFBb73	Big	–	–
CFBb10	Irregular	11°24′21.4″	107°24′25.6″	CFBb74	Big	–	–
CFBb11	Big	11°24′20.9″	107°24′22.3″	CFBb75	Small	–	–
CFBb12	Small	11°24′20.9″	107°24′22.1″	CFBb76	Irregular	20°20′56.7″	105°35′48.1″
CFBb13	Big	11°24′19.6″	107°24′22.1″	CFBb77	Big	–	–
CFBb14	Small	–	–	CFBb78	Small	20°15′51.2″	105°41′57.6″
CFBb15	Big	11°24′19″	107°24′22.2″	CFBb79	Big	20°20′56.6″	105°35′47.4″
CFBb16	Big	11°24′18.6″	107°24′22.3″	CFBb80	Small	–	–
CFBb17	Irregular	11°24′21.4″	107°24′21.4″	CFBb81	Irregular	–	–
CFBb18	Big	–	–	CFBb82	Big	20°15′7.6″	105°42′45″
CFBb19	Big	11°28′46.4″	107°22′52.9″	CFBb83	Small	–	–
CFBb20	Small	–	–	CFBb84	Big	20°14′53.5″	105°42′34.3″
CFBb21	Medium	11°23′18.7″	107°21′3.4″	CFBb85	Small	–	–
CFBb22	Medium	–	–	CFBb86	Small	–	–
CFBb23	Big	11°29′7.2″	107°22′58″	CFBb87	Medium	20°14′59.8″	105°42′28.1″
CFBb24	Small	–	–	CFBb88	Medium	–	–
CFBb25	Medium	11°27′56.1″	107°22′43.5″	CFBb89	Big	–	–
CFBb26	Big, yellow	11°27′0.9″	107°21′27.1″	CFBb90	Small	–	–
CFBb27	Small	–	–	CFBb91	Big	20°14′56.7″	105°42′21.1″
CFBb28	Big	11°27′1.5″	107°21′27.5″	CFBb92	Big	–	–
CFBb29	Small	–	–	CFBb93	Big	20°14′39.1″	105°42′25.3″
CFBb30	Big, red	11°26′56.6″	107°21′35.9″	CFBb94	Small	–	–
CFBb31	Big	–	–	CFBb95	Big	20°20′56.7″	105°35′46.5″
CFBb32	Big	11°23′18.8″	107°21′3.6″	CFBb96	Small	–	–
CFBb33	Small	–	–	CFBb97	Medium	20°20′58.4″	105°35′36.5″
CFBb34	Big	11°27′31.9″	107°20′43.1″	CFBb98	Medium	–	–
CFBb35	Big	11°27′5″	107°27′25.6″	CFBb99	Big	–	–
CFBb36	Irregular	11°27′3″	107°21′51.7″	CFBb100	Big	–	–
CFBb37	Small	11°27′5″	107°27′25.6″	CFBb101	Small	–	–
CFBb38	Small	–	–	CFBb102	Big	20°21′0.5″	105°35′36.3″
CFBb39	Big	11°27′17.9″	107°22′5.6″	CFBb103	Big	–	–
CFBb40	Big	11°27′17.9″	107°22′5.6″	CFBb104	Small	–	–
CFBb41	Small	11°27′17.9″	107°22′5.6″	CFBb105	Small	–	–
CFBb42	Big	11°26′47.8″	107°26′19.8″	CFBb106	Big	–	–
CFBb43	Small	11°26′47.8″	107°26″19.8″	CFBb107	Small	–	–
CFBb44	Big	11°27′50.4″	107°27′43.5″	CFBb108	Big	20°20′54.1″	105°35′48.5″
CFBb45	Irregular	11°27′51.8″	107°27′42.1″	CFBb109	Small	–	–
CFBb46	Big	–	–				
CFBb47	Small	–	–				
CFBb48	Big	11°23′19.3″	107°21′3.7″				
CFBb49	Small	–	–				
CFBb50	Big, pink	11°26′14.5″	107°25′24.2″				
CFBb51	Small	–	–				
CFBb52	Big	11°26′12.1″	107°25′25.7″				
CFBb53	Small	11°26′12.1″	107°25′25.7″				
CFBb54	Small	11°26′12.1″	107°25′25.7″				
CFBb55	Big	11°25′23″	107°25′41.4″				
CFBb56	Small	–	–				
CFBb57	Medium	11°23′26″	107°21′1.8″				
CFBb58	Big	11°24′49″	107°25′27.7″				
CFBb59	Big	–	–				
CFBb60	Small	–	–				
CFBb61	Small	–	–				
CFBb62	Big	–	–				
CFBb63	Medium	11°24′48.2″	107°25′25.9″				
CFBb64	Small	11°23′39.7″	107°21′47.9″				

Dashes indicate the same site above.

a Colonies had circular shapes and white color, otherwise noted as “irregular” and specific color.

**Table S4:** Counts of surviving and lifespan of C. elegans on *Microbacterium* sp. CFBb37 and the OP50 control.

**Day of lifespan**	**Number of surviving worms**	
	**OP50**	**CFBb37**
1	73	60
2	73	60
3	73	60
4	73	60
5	73	60
6	73	60
7	73	60
8	73	60
9	72	59
10	72	59
11	69	59
12	66	59
13	63	58
14	46	54
15	39	53
16	36	52
17	25	52
18	18	49
19	16	48
20	14	48
21	13	47
22	12	47
23	11	45
24	7	43
25	6	43
26	6	42
27	4	36
28	3	30
29	3	25
30	1	24
31	1	16
32	0	11
33		6
34		1
35		0
**Censored**	7	0
**Lifespan (days) (mean ± 1SE)**	17.26 ± 0.57	26.77 ± 0.86
*P* (log-rank test)		<0.001

SE, standard error.

**Table S5:** Counts of daily uterus eggs of close nematode species.

**Nematode**	**Reproductive day number**	**OP50**			**CFBb37**			**Different between the strains *P* (paired *t*-test)**
		**Number of tested hermaphrodites**	**Number of eggs in uterus/individual (mean ± SE)**	**Greater than zero *P* (one-sample *t*-test)**	**Number of tested hermaphrodites**	**Number of eggs in uterus/individual (mean ± SE)**	**Greater than zero *P* (one-sample *t*-test)**	
*C. elegans*	Day 1	32	3.94 ± 0.68	<0.001	35	1.57 ± 0.34	<0.001	0.0051
	Day 2	32	25.25 ± 1.12	<0.001	31	3.13 ± 0.32	<0.001	<0.001
	Day 3	29	5.38 ± 0.98	<0.001	42	4.02 ± 0.33	<0.001	0.06615
	Day 4	30	1.13 ± 0.34	<0.01	32	3.69 ± 0.33	<0.001	<0.001
	Day 5	37	0.03 ± 0.03	>0.05	34	3.5 ± 0.46	<0.001	<0.001
	Day 6				37	3.22 ± 0.51	<0.001	
	Day 7				34	2.29 ± 0.36	<0.001	
	Day 8				35	0.43 ± 0.16	<0.01	
*C. briggsae*	Day 1	39	3.30 ± 0.73	<0.001	39	0.51 ± 0.09	<0.001	<0.001
	Day 2							
	Day 3	34	4.09 ± 0.39	<0.001	35	0.6 ± 0.15	<0.001	<0.001
	Day 4	39	2.38 ± 0.24	<0.001	32	0.81 ± 0.14	<0.001	<0.001
	Day 5	35	0.2 ± 0.07	<0.01	35	1.46 ± 0.15	<0.001	<0.001
	Day 6	34	0.09 ± 0.05	>0.05	31	1.45 ± 0.19	<0.001	<0.001
	Day 7				32	0.63 ± 0.15	<0.001	
*C. tropicalis*	Day 1	33	2.09 ± 0.22	<0.001	32	0.28 ± 0.09	<0.001	<0.001
	Day 2	32	3.47 ± 0.23	<0.001	37	2.76 ± 0.32	<0.001	0.2149
	Day 3	38	1.87 ± 0.23	<0.001	36	3.19 ± 0.4	<0.001	0.0044
	Day 4	28	0.64 ± 0.23	<0.05	38	2.6 ± 0.24	<0.001	<0.001
	Day 5	36	0.19 ± 0.13	>0.05	34	2.94 ± 0.36	<0.001	<0.001
	Day 6				36	1.53 ± 0.2	<0.001	
	Day 7				27	1.63 ± 0.22	<0.001	
	Day 8				17	0.82 ± 0.16	<0.01	
*P*. sp.	Day 1	34	3.23 ± 0.55	<0.001	32	3.28 ± 0.26	<0.001	0.595
	Day 2	36	3.67 ± 0.63	<0.001	38	1.61 ± 0.18	<0.001	<0.001
	Day 3	39	1.38 ± 0.24	<0.001	36	1.58 ± 0.12	<0.001	0.629
	Day 4	36	0.19 ± 0.03	<0.05	36	1.56 ± 0.12	<0.001	<0.001
	Day 5	34	0.15 ± 0.03	>0.05	40	1.80 ± 0.16	<0.001	<0.001
	Day 6				35	1.06 ± 0.13	<0.001	<0.001
	Day 7				53	0.81 ± 0.10	<0.001	<0.001

SE, standard error.
